# Great ape gestures: intentional communication with a rich set of innate signals

**DOI:** 10.1007/s10071-017-1096-4

**Published:** 2017-05-13

**Authors:** R. W. Byrne, E. Cartmill, E. Genty, K. E. Graham, C. Hobaiter, J. Tanner

**Affiliations:** 1 Centre for Social Learning and Cognitive Evolution, School of Psychology and Neuroscience, University of St Andrews, St Andrews, Fife, KY16 9JP UK; 2 Department of Anthropology, University of California, Los Angeles, 375 Portola Plaza, 341 Haines Hall, Box 951553, Los Angeles, CA 90095 USA; 3 Laboratoire de cognition comparée, Institut de Biologie, Université de Neuchâtel, Rue Emile-Argand 11, 2000 Neuchâtel, Switzerland

**Keywords:** Gesture repertoire, Gesture meaning, Gesture ontogeny, Gesture phylogeny

## Abstract

Great apes give gestures deliberately and voluntarily, in order to influence particular target audiences, whose direction of attention they take into account when choosing which type of gesture to use. These facts make the study of ape gesture directly relevant to understanding the evolutionary precursors of human language; here we present an assessment of ape gesture from that perspective, focusing on the work of the “St Andrews Group” of researchers. Intended meanings of ape gestures are relatively few and simple. As with human words, ape gestures often have several distinct meanings, which are effectively disambiguated by behavioural context. Compared to the signalling of most other animals, great ape gestural repertoires are large. Because of this, and the relatively small number of intended meanings they achieve, ape gestures are redundant, with extensive overlaps in meaning. The great majority of gestures are innate, in the sense that the species’ biological inheritance includes the potential to develop each gestural form and use it for a specific range of purposes. Moreover, the phylogenetic origin of many gestures is relatively old, since gestures are extensively shared between different genera in the great ape family. Acquisition of an adult repertoire is a process of first exploring the innate species potential for many gestures and then gradual restriction to a final (active) repertoire that is much smaller. No evidence of syntactic structure has yet been detected.

Great ape communicative signalling has been a focus of investigation for over 60 years and never more so than at the present. The reason for this level of interest is clear enough: beyond the intrinsic value of understanding the natural signalling of any animal species, the communication of the great apes (hereafter, apes) holds out the promise of understanding the evolutionary origin of human language (Fitch 2010), often cited as our greatest cognitive distinction from other animal species (Wallman 1990). Language is an immensely complex system, found universally among human groups despite vast cultural differences, and the idea that this entire system could spring into being in a few million years of independent evolution lacks plausibility (Dawkins 1986; Tomasello 1995). Only by tracing precursors to language among our closest relatives are we likely to dispel the appearance of magic that is produced by setting human language in contrast to the “languages” of most animal species, however fascinating each may be to the biologist. Research on primates is sometimes castigated for taking an overly anthropocentric approach: in the case of ape communication, no apology need be made for an explicit approach of sometimes comparing directly to aspects of human language (Fitch 2010). That is what needs to be done.

## Ape gestures are intentional signals

For several reasons (including the predominance of speech in human communication, and the early availability of devices for electronic playback and spectrographic analysis), research on primate communication has concentrated on the vocal medium for much of the last 60 years. Ape vocalizations are highly graded, making identification of unit signals difficult and slowing progress (Marler and Tenaza 1977). Indeed, the evidence from apes still seems meagre, when compared to what is now known about the more discrete monkey vocalizations; for instance, it is only in the last few years that any vocalization meeting the criteria for “functional reference”—originally identified over 35 years ago in monkeys (Seyfarth et al. 1980)—has been described in apes (Schel et al. 2013a). However, it is great apes that have provided the only evidence to date that *any* primate vocalizes in a goal-directed, intentional way (Crockford et al. 2012; Schel et al. 2013b). When realistic model snakes are revealed by experimenters, chimpanzees aware of the “danger” target their warning calls at allies who were not present when the snake model was moved, and who are thus likely to be ignorant of the risk—unlike the indiscriminate broadcast of monkey alarm calls (but see Wich and de Vries 2006). For monkeys, and indeed almost all members of the animal kingdom, their natural communication has not required researchers to invoke an individual’s intention, only the adaptive value of giving a signal in specific circumstances (Seyfarth and Cheney 2003). They just do it; they do not have a plan in mind.

Against this background, the discovery that ape gestures are routinely given in an intentional way was a remarkable one. Tomasello and his collaborators studied chimpanzees in captivity and documented their natural gestural repertoire for the first time, finding that many gestures were given intentionally (Tomasello et al. 1985, 1989, 1994). That is, a chimpanzee would typically wait briefly after gesturing (“response waiting”), continuing to monitor their audience to assess the behavioural outcome; if no result was forthcoming, they would persist in gesturing, and if their audience had apparently not seen them, they would move round in front of them before persisting in gesturing (Liebal et al. 2004b). Leavens and Hopkins investigated intentional chimpanzee communication in greater detail (Leavens and Hopkins 1998; Leavens et al. 2005), demonstrating experimentally that chimpanzees, shown a desirable food, would persist and elaborate their gestural signalling if their keeper was reluctant to give them the whole of it, but never if they got what they wanted. The signals were targeted at a specific audience, to produce a specific behavioural result. We took this design one step further, working with orangutans (Cartmill and Byrne 2007), and investigated whether the apes would distinguish between a keeper who apparently misunderstand their gestural signalling (giving them an unwanted food type), versus one who partly understood (giving them half of the desired food). They did: with “partial understanding” by the keeper, orangutans persisted with the same types of gesture, increasing the rate; faced with “complete misunderstanding” they persisted in gesturing, but switched to different gesture types. Thus, apes—or at least orangutans, since this experiment has not been repeated with other species—continually monitor the communicative situation, not only to judge whether they have achieved their intended goal, but also to assess the level of understanding of their audience in order to maximize the effectiveness of their persistent gesturing.

Appropriate targeting of an audience is shown in other ways. Gestures vary in modality: some involve contact with the recipient’s body, so can be detected by tactile sensation even in an inattentive audience. Others do not, but produce an audible sound which may attract the attention of the audience to notice the gesture, or may be interpretable even without looking. Others are silent, and the audience’s visual attention would be required for effectiveness. Ape signallers show they are sensitive to these differences: for instance, chimpanzees and bonobos are more likely to use audible or silent visual gestures with an audience facing them, whereas for contact gestures no such effect is found (Call and Tomasello 2007b; Pika 2007). In the wild, we found that chimpanzees were more likely to use a silent visual gesture with an audience who was actually looking at them, and more likely to use a contact gesture with one who was not attending (Hobaiter and Byrne 2011a). Audible gestures showed no such variation: presumably since the audience should get the message whether or not they are attending visually.

The hallmarks of intentional usage have been found in bonobos, gorillas and orangutans, as well as chimpanzees (Call and Tomasello 2007a). Indeed, evidence of intentionality is abundantly shown in the everyday behaviour of apes, with signallers showing audience targeting, response waiting, and persistence and elaboration in cases where the target audience fails to react. Even insisting that each single *instance* of gesture use shows at least one of these criteria barely halves the corpus of gestures available for analysis (Genty et al. 2009), leaving thousands of cases for study. Since discovering that this was so feasible, we have used that criterion in all subsequent studies. On the other hand, intentional usage may not apply to facial expressions. Facial expressions, like vocalizations, do show audience effects: for instance, orangutan playfaces were more complex when given to a play partner who was facing them (Waller et al. 2015). However, the level of voluntary control of facial expression seems limited, compared to gesture use (Darwin 1872; Porter et al. 2012). Tanner and Byrne (1993) showed that a gorilla, intent on a game of surprising her reluctant play partner, developed a technique of hiding or wiping off her revealing “play face” expression as she approached him; it seems that “leakage” of motivational state could not be inhibited when it affected the play face, whereas the hands were under greater voluntary control. This difference in their intentionality means that it is safer to analyse facial expression and gesture as independent systems.

We should stress that none of the evidence for intentional gesture (or vocal) usage by apes goes beyond first-order intentionality (Townsend et al. 2016); that is, it is evidence that a signaller has a specific result in mind, in terms of another individual’s behaviour, and will work flexibly to achieve that result. There is no evidence to date that ape signallers intend to change the knowledge or beliefs of their audiences. Whether this distinction simply reflects the difficulty of obtaining convincing evidence of second-order intentionality in naturally observed gesture, or marks a real limit on ape mentalizing, is not yet known. The evidence, mentioned above, that orangutans can assess their audience’s level of understanding hints at the former.

## Repertoires are large and extensively shared between ape species

The first studies of ape gesture reported relatively small repertoires, based on single-site captive studies (e.g. gesture counts: chimpanzee 26: Tomasello et al. 1985, 1989, 1994, and 31: Pollick and de Waal 2007; bonobo 21: De Waal 1988, and 24: Pika et al. 2005; gorilla 36: Tanner 1998, and 33: Pika et al. 2003; orangutan 29: Liebal et al. 2006). As more wide-ranging studies were conducted, able to assess a greater range of social circumstances by including different captive groups or by using field study, much larger repertoires emerged (e.g. chimpanzee 66: Hobaiter and Byrne 2011a; bonobo 68: Graham et al. 2016; gorilla 102: Genty et al. 2009; orangutan 64: Cartmill and Byrne 2010).

In evaluating these numerical estimates, it must be remembered that definition of “a gesture” may vary between researchers: for instance, Tanner (1998) restricted attention primarily to manual gestures, whereas Genty et al. (2009) included many communicatory body postures and movements. More fundamentally, researchers have typically based their definitions on physical form, so the question arises of the appropriate granularity of description: the “right” level of splitting or lumping (Cartmill and Byrne 2011). We believe that this should be settled by the apes themselves, by using the gestures’ meanings: as would be done when compiling a lexicon of words. Beginning at the lowest (most fine-grained) level of categorization, physically similar gestures can be lumped if signallers’ intended meanings do not differ significantly; in the case of the chimpanzee, this procedure resulted in both splits and lumps compared with previous classification based only on gesture form (Hobaiter and Byrne 2017). In addition, greater comparability can be achieved by working together and closely sharing criteria. Since we have now worked with *Pongo*, *Gorilla*, *Pan troglodytes* and *Pan paniscus*, using essentially the same criteria for gesture definition, we are in a strong position to do so: Table 1 shows the results of these “St Andrews’ studies”. Details aside, it is clear that apes have very extensive gestural repertoires.

**Table 1:** St Andrews Catalogue of great ape gestures

Gesture	Description	Contains	PTSch	PPan	Gor	Pon
Arm(s) out^a^	Extend arm(s) out horizontally from the shoulder			+		
Arm(s) raise	Raise hand(s) or arm(s) vertically above shoulder	Arm(s) raise with object, arm(s) up, hand(s) raise, raise arm(s)	+	+	+	+
Arm(s) shake	Small repeated back and forth motion of arm(s)	Arm(s) shake on, arm(s) shake with object	+	+	+	
Arm(s) swing	Large back and forth movement of arm(s) from shoulder	Arm(s) swing direction, arm(s) swing under, arm(s) swing with object, down, up	+	+	+	
Arm(s) wave	Large back and forth movement of arm(s) raise above shoulder	Arm(s) wave with object; Straw wave	+	+	+	+
Beckon	Hand moved in a sweep from elbow or wrist towards signaller	Beckoning, finger curl	+	+	(+)	+
Big loud scratch	Loud exaggerated scratching movement on signaller’s own body	Self-scratch	+	+		+
Bipedal rocking^a^	Side to side or forward and back movement while standing/walking bipedal (rarely also quadrupedal)	Swagger	+	+	+	
Bipedal stance	Standing bipedally, arms often held out to side with back arched		+	+	+	
Bite	Recipient’s body is held between or against lips or teeth of signaller	Kiss, mouthing/gnawing, open mouth kissing, submissive kissing	+	+	(+)	+
Body drum	Signaller slaps body with hand(s) to make contact	Armcross, beat sides of head, body beat, body beat with object, chest beat play, chest pat, drum belly, slap cheek, slap shoulders			+	
Bounce^a^	Up and down movement of whole body flexing elbows or knees, typically while quadrupedal		(+)	+	+	
Bow	Signaller bends forward from waist while bipedal	Bow-extend, bowing	+	+	+	
Chest beat	Signaller slaps chest with cupped hand(s) to make loud audible contact	1-handed chest beat			+	
Clap	Palms of both hands or feet brought together with audible contact	Clap hands, feet clap, hand clap	+	(+)	+	+
Dangle	Signaller hangs from arm(s) above another, may shake feet/legs, typically audible with movement in canopy	Dangle with feet shake, rope spinning, rope swinging, swing	+	+	+	+
Disco arms shake	Shaking arms in rotation movement towards self	Circle hands			+	
Drum object	Short hard audible contact of alternating palms against object	Drum, drum object fists/palms, slap ground	+		+	
Drum other	Short hard audible contact of alternating palms against recipient		+		+	
Embrace	Signaller wraps arm(s) around recipient and maintains physical contact	Embrace full, embracing, mounting	+	+	+	+
Feet shake	Small repeated back and forth motion of feet or leg(s)	Legs shake	+		+	
Finger in mouth^a^	Finger(s) are placed into the mouth of the recipient		(+)			
Gallop	Exaggerated running movement where contact of hands and feet is deliberately audible	Gallop with object, stiff gallop	+	+	+	
Grab	Signaller hand(s) is firmly closed over part of recipient’s body	2-handed grab, air grab, hair pulling, hands around head, head-grab, face-grab, grab-hold, grasp, restrain	+	+	+	+
Grab-pull	As grab but closed hand(s) contact maintained and a force exerted to move recipient from current position	2-handed grab-pull, hand in neck, hand leading, pull, pull away, pull face to face, pull hair, pull towards, lead	+	+	+	+
Hand(s) fling	Rapid movement of hand(s) or arm(s) from the signaller towards the recipient	Arm threat, away, go, hand wave off, hitting away, flap, flapping, raise arm quickly, shoo	+	+	(+)	+
Hand(s) on	Hand—typically palm or knuckles—placed on recipient with contact lasting >2 s	Arm on, pat off	+	+	+	+
Hand(s) shake	Small repeated back and forth motion of hand(s) from wrist	Hand(s) shake with object, shake wrist	+	+	+	
Head butt	Head is briefly and firmly pushed into recipient’s body	Head on	+	+	+	
Head rub	Back and forth movement of palms of hand(s) over the signaller’s head				+	
Head shake	Small repeated back and forth motion of head	Bob, chin up/nod, head bob, head nod, head rock, head shake with object, head tipping, head turn, head twirl, tip head	+	+	+	+
Head stand	Signaller bends forward and places head on ground		+	+	(+)	+
Hide	Body part, e.g. face, genitals, is hidden by the hand(s) or arm(s)	Cover, hide face, hide playface	+		+	+
Hip thrust^a^	Sitting, crouching, or standing, the hips are thrust forward (single or repeated)	Thrust	+	+		
Hit with object	Signaller brings object into short hard contact with recipient’s body	Club	+	+	+	
Jump	While bipedal both feet leave ground simultaneously with horizontal displacement	Bipedal jump	+	+	+	
Kick	Foot/feet brought into short hard contact with recipient’s body with horizontal movement	Kick backwards	+	+	+	
Knock object	Back of hand/knuckles brought into short hard audible contact with object	Knock, rap, rap knuckles	+	+	+	
Leaf clip	Strips are torn from a leaf/leaves using hand or mouth, making a conspicuous rhythmic sound	Clip leaf	+			
Leaf drop	A leaf(s) is picked off and dropped, usually signaller is above recipient	(note: similar usage to leaf clip)		+		
Leg(s) flap	Sitting with knees bent, one or both legs opened and closed to the side (single or repeated)			+		
Leg(s) rub	Back and forth movement of palms of hand(s) over the signaller’s leg(s)				+	
Leg(s) swing	Large back and forth movement of leg(s) from hip		+	+	+	
Lick hand	Licking of the palm frantically and repetitively				+	
Look	Signaller holds eye contact with recipient lasting >2 s	Peer, peering, putting face close, look back, wait	+	+	+	+
Mouth stroke	Signallers palm or fingers repeatedly run over mouth area of recipient	Hand beg, rub chin	+	+		
Object in mouth	Signaller approaches recipient while carrying object (e.g. small branch) in the mouth		+		(+)	
Object move	Object is displaced in one direction, contact is maintained with object throughout	Branch dragging, drag branch, push backwards, push object, rake/scratch dead leaves, scrub, sway vegetation	+	+	+	+
Object on head	Object is placed on head		(b)	(b)	+	+
Object shake	Repeated back and forth movement of an object	Branch shaking, branch rinse, flail, shake detached branch, shake object	+	+	+	+
Pirouette	Signaller turns around their body’s vertical axis	Ice skating, Pirouette with object	+	+	+	
Poke	Firm, brief, push of one or more fingers into recipient’s body, may be repeated	Poke at, poking, hard touch, tickling and poking	+	+	+	+
Pounce	Signaller displaces through air to land quadrupedally on the body of the recipient		+	+	+	
Present body part	Body part is moved to deliberately expose an area to recipient’s attention	Back offer, belly offer, foot back, foot present, present climb-on, present groom, flexed knees, leg bending, lead forward, lie with back to another, lower/raise leg, lowering back, solicit grooming, turn face downwards	+	+	(+)	+
Present genitals	Genitals are moved to deliberately expose them to recipient’s attention	Present, present genitals forwards/backwards, present rear, present with limbs flexed	+	+	+	+
Punch object/ground	Movement of whole arm, with short hard audible contact of closed fist to an object or the ground	Backhand, thump	+		+	
Punch other	As punch object/ground but contact is with recipient’s body	Hit, wrist hit	+	+	+	
Push	Palm(s) in contact with recipient’s body and force is exerted in attempt to displace the recipient	Back push	+	+	+	+
Push directed	A light short non-effective push that indicates a direction of desired movement, immediately followed by the recipient moving as indicated	Direct-hand, positioning, turn head, tactile close gestures	+	+	+	+
Reach palm	Arm is extended to the recipient with hand in open, palm exposed position (no contact)	Beg, begging with hand, extend hand, extend palm, holding hard towards another, reach, reach hand, stretch out hand,	+	+	+	+
Reach wrist^a^	As reach palm, but wrist or back of hand extended towards recipient with palm in sheltered position	Offer arm, reach, stretch out hand, wrist bending	+	+	+	+
Rocking^a^	Large back and forth movement of body while seated or quadrupedal		+	+	+	+
Roll over	Signaller rolls onto their back exposing stomach, can be accompanied by repeated movements of arms and/or legs	Lie down on back	+	+	(+)	+
Rump rub	Rump area is pushed and/or rubbed with small repeated up and down movements against the body of the recipient	Rump turn	+	+		
Shake hands	Signaller grasps recipient’s hand/fingers in their own hand and makes small repeated back and forth movements from the wrist	Hand holding/shaking, hold hand	+	+	(+)	+
Show	Arm holding object is partially extended towards recipient and held					+
Shrug	Shoulder is raised quickly against recipient					+
Side roulade	Body is rotated around the head-feet axis while lying on the ground		+	+	+	
Slap object/ground	Movement of the arm from the shoulder with hard short contact of the palm(s) to an object or the ground	Ground slap, hit ground/object, slap surface, slap–stomp	+	+	+	+
Slap object with object	As slap object but the hand(s) which is brought into contact with an object holds another object	Banging, club ground	+	+	(+)	
Slap other	As slap object/ground but contact is with recipient’s body	Club another, hit, poke at, simultaneous hit, slap other with object, slapping	+	+	+	+
Somersault	Signaller’s body is curled into a compact position on the ground and rolled forwards or backwards so the feet are brought over the head	Back roll	+	+	+	+
Stiff stance	Standing rigidly with still limbs and forelimbs held tight, usually with facial expression of tight lips				+	
Stiff walk	Walk quadrupedally with a slow exaggerated movement	Play walk, stiff 3-feet walk	+	+	+	
Stomp	Sole of the foot/feet is lifted vertically and brought into short hard contact with the surface being stood upon	Foot beat, heel kicking, multiple stomp, multiple stomp 2-feet, stamp, stamp 2-feet, stamp object, stamping, stomp 2-feet, stomp ritualized, stomping	+	+	+	
Stomp other	As Stomp but contact is with recipient’s body	Foot stomp, jumping, stamping on the back, stomp other 2-feet	+	+	+	
Stroke^a^	Stroking another individual with gentle back and forth movement of the palm(s) or fingers	Brush, Stroking	+	+	+	+
Tandem walk	Subject positions arm over the body of the recipient and both walk forward while maintaining position	Arm neck, arm round, embrace half	+	+	+	+
Tap body	Movement of the arm from the wrist or elbow with firm short contact of the fingers to the signaller’s body (may include rhythmic repetition)	Body tapping with object, single body tap, tapping body, tapping contralateral			+	
Tap object	As tap body but contact is with object	Tapping object	+	+	+	
Tap other	As tap body but contact is with recipient’s body	Tap, tapping other	+	+	+	+
Throw object	Object is moved and released so that there is displacement through the air after release	Aimed throwing, drop branch, lift and drop, throw at, throw threat	+	+	+	
Touch other	Light contact of the palm and/or body of the recipient, for under 2 s	Hands on shoulders, hold, light touch, touch, touch-side	+	+	+	+
Water splash	Hand or feet moved vigorously through the water so that there is audible displacement of water	Hit water	+	+	+	

+ indicates that the gesture is present with video evidence of intentional use in our group, (+) indicates that the gesture is present but without existing video evidence of intentional use in our group, (b) indicates that the action is seen in that species but with no observations of its intentional use in our group. Gorilla gestures only produced by single captive individuals with a close history of human interaction were excluded here, but these included: face wipe, finger down lips, hand(s) between legs, hands behind back, mouth/lips, teeth, wrist glance

PTSch, *Pan troglodytes schweinfurthii*; PPan, *Pan paniscus;* Gor, *Gorilla gorilla*; Pon, *Pongo*

^a^A new gesture type as compared to our previous published catalogues for gorillas (Genty et al. 2009; Tanner and Byrne 1999; Tanner 1998), chimpanzees (Hobaiter and Byrne 2011a, b) and bonobos (Graham et al. 2016)

In early work, a high degree of idiosyncrasy was reported. To some extent, that impression was a matter of definition: a gesture was considered idiosyncratic if only one individual in a small group used the gesture during a relatively short study period, even if others had used it during other periods before or since (Tomasello et al. 1994). Yet even with a more conventional understanding of idiosyncrasy, certain gestures appeared “particular” to certain individuals. Once more, the picture changed with increasing evidence. In gorillas studied at four different zoos and one field site, only one gesture type among 102 was found to be idiosyncratic, and that was used specifically to a human keeper (Genty et al. 2009). In chimpanzees studied at Budongo, Uganda, no idiosyncratic gesture was recorded at all (Hobaiter and Byrne 2011a). Moreover, the Budongo repertoire was found to include almost all gestures reported at other chimpanzee field sites; admittedly, those studies were not specifically of gesture, but they extended over very long periods. Indeed, the level of overlap between all chimpanzee studies—captive and field—was found to be so high that, to a first approximation, the repertoires could be described as the same (there was a small number of site-specific exceptions, but several of those have since been described at other sites: the approximation becomes increasingly accurate). Data from Budongo chimpanzees have also revealed a good reason why an initial impression of idiosyncrasy was found (Hobaiter and Byrne 2011a). When the estimated repertoire was plotted against observation time, the community repertoire rose to an asymptote, giving confidence in the final total. However, when individual chimpanzees were mapped onto the same axes, all individuals fell well below that asymptote, lying instead in the steeply increasing part of the curve (Figure 1 of Hobaiter and Byrne 2011a). Indeed, observation time was shown to be a strong predictor of an individual’s “repertoire”. If the repertoire of every subject was seriously underestimated in an 18-month field study, it is unsurprising that a misleading appearance of idiosyncrasy is seen when repertoires are compared after much shorter periods. Ape repertoires are so large, with many gestures used only occasionally or at certain stages of life, that intensive and prolonged study is needed to come close to an individual’s true repertoire.

**Fig. 1:**
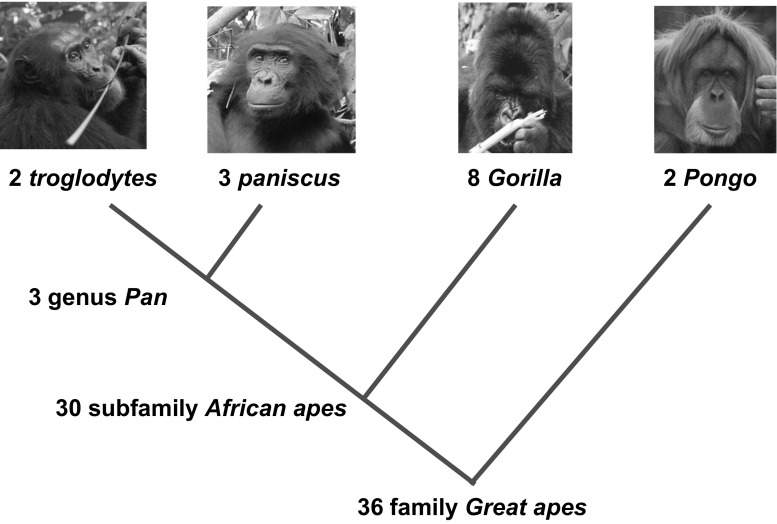
The distribution of gestures across living great ape species and genera, based on current knowledge: numbers of gestures specific to each clade are shown, revealing extensive overlap at higher taxonomic levels. Where a gesture is found in all of *Pongo*, *Gorilla* and *Pan* it has been treated as ape-typical even if it has not yet been recorded in both *troglodytes* and *paniscus*. Note that one gesture, *big loud scratch*, appears to have been lost in the genus *Gorilla*, although it is of course difficult to be sure of absence

More striking still, repertoires have been found to overlap *across species*, and even genera, of ape (Fig. 1). The level of taxonomic overlap in gesture forms is striking, and more so, when it is remembered that the apes differ widely in hand structure and mode of locomotion. *Gorilla* has short fingers and relatively long thumb, like *Homo*, whereas *Pan* has long fingers and short thumb; *Pongo* travels by suspensory “4-handed” clambering, *Pan* and *Gorilla* by knuckle walking and manual brachiation. (Note that, as evidence accrues, just as in the case of the vanishing idiosyncrasy, apparent differences between species and genera are liable to be revealed as false, resulting from imperfect sampling.) A possible explanation of overlap in gesture form might of course be that there is little choice: when making 70–90 different gestures that are sufficiently distinct from each other, the natural constraints of hands and body might force similar gesture types in all apes. On statistical grounds, however, this is unlikely. By identifying the dimensions on which actual ape gestures differ—their “morphological features”, as it were—we constructed a set of all possible ape gestures (Hobaiter and Byrne 2017). When all those that are physically impossible had been excluded, we were still left with over 1000 entirely possible gestures. That all three genera have converged on closely similar sets of gestures among all the 1000+ possibilities is unlikely to be coincidence. A simple and parsimonious explanation is that of common descent: the potential to make the gestures of each species’ repertoire is innate and thus heritable. This interpretation remains disputed (Halina et al. 2013), but at present we consider that the burden of proof should be on those who favour an individual-learning account to provide clear evidence in its favour.

## Ontogeny is (largely) phylogenetic

The first hypothesis for gesture ontogeny to be investigated was that of learning from conspecifics, as in language (Tomasello et al. 1994). However, when individual repertoires were compared between and within communities, the degree of similarity was the same, quite contra to the local dialects that would be expected with social transmission of gesture types. Social learning may be important in a limited way, as several gestures have been described as parts of chimpanzee “culture” on the basis of inter-site differences in occurrence (Whiten et al. 1999); alternatively, of course, those differences may reflect inadequate sampling of gestures that are relatively rare at some field sites.

The idea of ontogenetic ritualization was instead proposed (Tomasello et al. 1985; Tomasello and Call 2007), based on a hypothesis of Plooij (1978). On this hypothesis, the young ape first tries to achieve its aims by force: to get food, it reaches out to grab it; to climb on the mother, it raises two hands from below and holds on; and so on. The mother is able to interpret the “wants” that lie behind these actions, in advance of the full action, and in most cases is cooperative with her infant; she thus responds in anticipation, having seen only the first part of the infant’s action sequence (an “intention movement” in traditional ethological terms: Smith 1977). In turn, the infant comes to rely on that anticipatory reaction, thus is unintentionally tutored by its mother, and bothers only to begin the sequence: e.g. holding up both hands, to climb on the mother. At this point, the infant has acquired a gesture, which it can use instead of the physically effective action sequence it began with. Such a gesture will have very specific properties. Its usage will be intentional, since that is how it originated. Its form will be physically “like” some early aspects of the forceful action, and the exact form may be different for different pairs of tutor and learner. It will be “one-way”, in communicative force. That is, while the mother has unintentionally trained the infant to use an action as a communicative gesture, the reverse has not taken place; thus, the gesture is not part of a shared repertoire but functions only from infant to mother (or whoever the two individuals concerned). Since ontogenetic ritualization relies only on classical conditioning, there is no doubt that it could happen: in specific cases, dyadic learning of this kind has been shown to modify ape gestures (Halina et al. 2013). But does it account for the acquisition of the gestural repertoire as a whole? Does what is known of ape gestures in general match the characteristics expected from ontogenetic ritualization?

For a start, there is now less need for an explanation for idiosyncrasy, as little idiosyncrasy is found when in-depth studies are carried out; indeed, the lack of idiosyncratic gestures becomes a problem for the idea of individual learning of each gesture. Since repertoires are in the main species-typical and extensively shared even between species and genera, the alternative explanation of an inherited repertoire—in everyday terms the idea of “innate” gestures—is possible, and biologically more straightforward. Such has been the accepted explanation of signal repertoires in other animal species, as described first by Lorenz (1966): phylogenetic ritualization. The frequent resemblance, between the communicative gesture and the physically effective action for the same result, is neutral between these theories. Just as one might expect the conditioning process of ontogenetic ritualization to seize on some part of the effective action, so would the evolutionary process of phylogenetic ritualization be most likely to act on variation of that sort: form will often mirror function, in both cases. Ontogenetic ritualization was considered to result in gestures that were used intentionally; it was accepted that other ape gestures would be innate, but they would not be used intentionally (Tomasello and Call 2007). However, when we divided the repertoire of gorillas, and later chimpanzees, into those gestures whose form plausibly mirrored their function and those where there seemed no obvious relationship, we found that both sorts were used just as intentionally (Genty et al. 2009; Hobaiter and Byrne 2011a): there was no distinction to be made. Little attention has been paid to the prediction from ontogenetic ritualization of one-way gestures. Of course, in some cases the ritualization process might have occurred from A to B, and also from B to A, producing symmetry; but there should at least be many cases where it had not, or where the gesture form that was ritualized differed between A and B. These possibilities were examined explicitly in the bonobo, but no convincing evidence of one-way gestures was found (Graham et al. 2016). Gesture use and comprehension was symmetrical.

More generally, the sheer amount of “work” that each ape would have to do, to acquire a repertoire of 70–80 gestures that are understood by most of its social group, and conversely to learn the significance of each of their own gestures when made towards itself, makes explanation of all gestures as a matter of individual learning in dyadic social contexts rather implausible (Byrne 2016). In some cases, the necessary reinforcement history is very hard to imagine at all. For instance, consider how an infant chimpanzee might learn by ontogenetic ritualization to use the gesture of holding out its hand with the back of the hand towards the target and flicking the fingers towards them (*hand fling)*, when it wants the target to move away (Hobaiter and Byrne 2011a, 2014). The gesture resembles the physically effective action of a backhanded slap to the face: but is it plausible that an infant begins life by face-slapping adults? In short, the evidence for ontogenetic ritualization as an acquisition mechanism for most ape gestures appears weak. Recently, Fröhlich et al. (2016) proposed a modification of the theory, in which an exchange of social behaviour results in a shared understanding that can be generalized across individuals. They argue that variation in the gestures employed for similar goals by individuals both within and between groups cannot be achieved by a biologically inherited repertoire of signals. However, this seems to stem from a misunderstanding of the phylogenetic argument. Phylogenetic ritualization limits the potential repertoire of available gesture types. Within that very large set of signals, the subset of gestures employed on a regular basis may be fine-tuned by social interaction (Hobaiter and Byrne 2011b), as is seen in the tuning of phonemes in human language (Oyama 1976). Thus, recent studies that highlight the importance of social interactions in the development of gesturing (see for example: Bard et al. 2013; Fröhlich et al. 2016; Hobaiter and Byrne 2011b; Schneider et al. 2012) are not incompatible with a phylogenetically ritualized set of available gesture types.

Tanner et al. (2006) have proposed that great apes possess a powerful mechanism, akin to the “intermodal matching” that Meltzoff and colleagues (Meltzoff and Moore 1977; Meltzoff and Prinz 2002) have suggested to underlie human infant imitation. They suggest that apes can represent mentally, and then enact through a kind of mime, the actions of others. On this “action mapping” hypothesis, gestures like *hand fling* might originate as enactments of the motion the signaller would like their audience to follow. However, given the limited abilities of even adult apes to mime (Russon and Andrews 2011), stronger evidence would be needed to accept such a powerful mechanism for the origin of ape gestures.

In contrast, it is not only simpler to view each species repertoire as largely determined by biology but this also explains the available facts well: accounting for the phylogenetic distribution of shared gestures shown in Fig. 1. While ape gestures are in a sense innate, that should not be misunderstood to mean present at birth, rigid and inflexible, or immune from developmental effects: any more, say, than is human bipedal walking. Nor does this preclude the possibility that *some* gestures may be learnt: either socially, by copying the form of gesture used by others as a social tradition, something chimpanzees at least are able to do (Byrne et al. 2011; Hobaiter and Byrne 2010); or by ontogenetic ritualization (Halina et al. 2013), a process which may be particularly likely in captivity when apes have excessive time on their hands. In the main, however, an ape develops its communicative repertoire of gestures by exploring its own innate potential to make a large range of different gestures for a range of different purposes. The result is a communication system in which any member of an ape community can make any of the gestures typical of its species.

## Gesture meanings are shared

“Meaning” is a loaded term when discussing animal communication. Normally, in order to avoid unwarranted imputation of goals to signallers, biologists describe signals by their function: the effects they produce on audiences and the fitness benefits of these effects for the signaller (Evans et al. 1993; Gaunet and Deputte 2011). But since the intentionality of ape gesture has been robustly established, it is appropriate to ask what signallers *mean*: what effects do they *want* to produce? In play, of course, gestures are not necessarily used with the same meaning (Fagen 1981; and see Tanner and Perlman 2016, for a recent analysis of gesture use in play). Indeed, most gestures have as one of their meanings a function specific to the modulation of play: initiating play, escalating or tempering its intensity (Cartmill and Byrne 2010; Genty et al. 2009; Hobaiter and Byrne 2014). It is therefore important, when seeking to find a gesture’s normal meaning, that data are used from non-playful contexts only. In practice, data from wild animals are therefore likely to be of the greatest value, since the lives of healthy captive apes are liable to be dominated by playing or resting.

To investigate intended meaning it is insufficient simply to measure effects, some of which may have been unintended consequences or even deliberate rebuffs of the signal. In order to exclude those potentially confusing responses, we select cases for analysis only where the target audience’s response was accepted as apparently satisfactory by the signaller: something we can judge by their cessation of signalling (Cartmill and Byrne 2010; Genty et al. 2009; Hobaiter and Byrne 2014). The accumulation of such responses is treated as a gesture’s “Apparently Satisfactory Outcome” (ASO), an operationalized version of the gesture’s meaning (note that some cases are sure to occur where the signaller simply gives up trying. These will give rise to false indications towards an ASO. We must therefore expect some spurious, low frequency “ASOs” to occur as background noise and only recurring patterns can be relied upon).

In principle, it would be possible for a community of apes to have gestures with individually specific meanings, such that an audience would need to know who was making a gesture to discern its meaning. In practice, the evidence is against that possibility: individual identity does not interact with gesture meaning (chimpanzees: Hobaiter and Byrne 2014; bonobos: Graham et al. 2016). A more realistic concern is that certain signals might be made by one age-sex class and directed at another, and indeed this is the typical case in many animal communication systems. The ability to make a specific gesture could be found in some individuals, and the ability to understand it in others. This pattern might be regarded as a special case of “one-way” gesture use, mentioned already, and our evidence from bonobos is relevant (Graham et al. 2016). For each gesture type, we recorded cases where individuals used or reacted appropriately to it. Then we categorized individuals as male versus female, and adult versus juvenile. When less than 3 cases were recorded overall, the data were discarded; in almost every remaining case, gestures were found to be *both* used *and* understood by each social grouping. There is thus every reason to think that ape gestures form a mutually understood communication system: all members of a community have the potential to make and understand all the many species-typical gestures in appropriate circumstances.

Even though the physical forms of gestures are extensively shared among ape species, it might be the case that their meanings differ among species. To investigate that possibility, we compared the meanings of gestures used by both chimpanzee and bonobo (Graham et al. in prep-b). For each gesture type, we recorded which of all possible outcomes apparently satisfied signallers and which did not: the degree to which these were the same in both species gave an index of similarity in usage. Then we generated 10,000 random assignments of gestures to meanings, with the constraints that each must have the same number of meanings per gesture and the same number of gestures per meaning as the real data. Chimpanzees and bonobos were significantly more similar than expected from this randomization test in how they assigned gestures to ASOs: indeed, not a single pairing of random assignments gave a value as high as the actual similarity between the two species. This implies that the gestural communication system is a common one across these closely related apes. Given the extensive sharing of physical forms of gestures with other genera, in *Gorilla* and even the relatively distantly related *Pongo*, it seems likely that assignments of meaning to gesture forms will also prove to be shared generally among the apes.

## How gestures convey meaning

For all ape species, the repertoire of gestures is much larger than the number of meanings (ASOs) that have been identified (non-play ASOs: orangutan 5: Cartmill and Byrne 2010; gorilla 10: Genty et al. 2009; chimpanzee 15: Hobaiter and Byrne 2014; bonobo 14: Graham et al. in prep-b). Thus, either we have simply failed to differentiate meaning at a fine enough level, and in reality there are many more shades of meaning, each unambiguously conveyed by a particular gesture; or, some gestures are redundant. The fact that apes, when confronting difficulty in achieving their intended meaning (e.g. a keeper deliberately “failing” to understand the intentions of an ape, as part of a planned experiment: Cartmill and Byrne 2007), readily substitute different gesture types, suggests that the lexicon of gestures is genuinely redundant. The degree of redundancy varies: in chimpanzees, some purposes are achieved regularly with single gesture types, whereas others apparently require several types. Hobaiter and Byrne (2014) noted that the latter seemed often to be in cases where there was no canonical response (e.g. when requesting affiliation), whereas in cases where the appropriate response was obvious (e.g. in grooming initiation) only a single gesture type was employed. They suggested that the redundancy of gestures might be helpful in situations requiring negotiation. Whether this conjecture will stand up to further analysis, and apply in other species, is not yet known.

In many cases, a single gesture appears to have more than one meaning: even when play data are excluded, it is typical to find two or more ASOs associated with a single gesture (Cartmill and Byrne 2010; Genty et al. 2009; Graham et al. in prep-b; Hobaiter and Byrne 2014). Since we have identified more gestures than ASOs, this apparent ambiguity is puzzling. However, it may be that the ambiguity is not apparent to the apes themselves, just as we seldom notice word ambiguity in normal speech. Consider the spoken word /ba:rk/. If we have taken our pet dog to the vet, we hear the word to mean a vocalization; if we are contemplating damage to a prized tree by a careless driver, we hear the word to mean tree epithelium; in discussing merchant marine history, the same word might be heard as a ship (barque). We are not aware of working out which of the ambiguous meanings is meant: indeed, most people are unaware that so many everyday words *are* lexically ambiguous (Vitello and Rodd 2015). Might context also aid disambiguation of ape gestures? For bonobo gesture types that showed several ASOs, we examined the distribution of ASOs across different interpersonal and behavioural contexts. For every gesture, the distribution of ASOs was significantly different in different contexts, with ambiguity of intended meaning almost completely removed in context (Graham et al. in prep-a). If we were to compare an ape’s gestural lexicon with the words of a language user, then these data would imply that each of the non-playful ASOs reflects gesture homonyms, thus considerably increasing the size of the ape gestural repertoire.

The intended meanings that gestures signal are, in the main, fairly prosaic (Cartmill and Byrne 2010; Genty et al. 2009; Graham et al. in prep-b; Hobaiter and Byrne 2014). Our labels for ASOs give the idea: “acquire object” causes the object to be given; “follow me” causes the signaller to be followed; “climb on me” causes an infant to climb on an adult carer’s body; “sexual attention (to male)” causes a female to respond sexually to the signaller; “stop that” causes cessation or change in current behaviour, and so on (all examples taken from chimpanzee lexicon: Hobaiter and Byrne 2014). A few intended meanings involve outcomes that are specific to locations: “reposition body” causes the target to move into and hold the indicated position; “attend to specific location” causes the target’s attention to focus on the indicated location. Such gestures have several times been claimed to be referential or iconic (Genty and Zuberbuehler 2015; Pika and Mitani 2006; Tanner and Byrne 1996), but care needs to be taken with those labels. None of the gestures can be understood without the additional information of the location at or towards which they are made: thus, deictic would be a more appropriate term than referential. Moreover, these gestures do not involve distal pointing, so it is not clear that the apes need to understand the deictic relationship between gesture and intention. For instance, it is reported that chimpanzees of the Ngogo community, Uganda, respond to the *directed scratch* of a body part by then grooming the signaller in that place (Pika and Mitani 2006): but does the observer understand the action as a kind of pointing, or has its attention simply been drawn to the site? The form of the gesture may physically resemble the movement pattern that the gesturer intends the target to make: e.g. the *armswing under* of a gorilla follows the path of the intended movement towards mating by the partner (Tanner and Byrne 1996), and the beckoning gesture of a bonobo, like the equivalent human gesture, follows the desired movement vector of the target (Genty and Zuberbuhler 2015). But are these gestures correctly interpreted by the apes because they understand the mimetic aspect of the movements (Russon and Andrews 2011)—realizing that they depict desired motion—or do they simply know what they mean? The case of the numerous English words that are onomatopoeic in origin may be analogous. Most speakers and hearers have no knowledge of the words’ onomatopoeic origin and simply know what they mean; the same is possible for iconic gestures in apes. That is, the phylogenetic origin of the gesture may indeed be based on a physical resemblance to the signaller’s intention to guide the mating partner in a desired movement path (Lorenz 1966), but that resemblance may be opaque to current users.

## Gesture sequences

All ape species sometimes produce gestures in series, as well as making them singly; several studies have examined whether structured conjunctions of gestures modify or change the meanings of individual gestures (Genty and Byrne 2010; Hobaiter and Byrne 2011b; Liebal et al. 2004a). The results have been uniformly negative. No convincing report has been made of any syntactical change of meaning based on co-occurrence with another gesture, and gestures given in a series have the same individual meaning. Two different explanations have been offered for gesture series, and both may be correct for series of different composition. Since gestures are used intentionally, yet target audiences may be unresponsive or reluctant, persistent gesturing is to be expected. If an ape makes a gesture, waits for a response, and—when none is forthcoming—gestures again, researchers may record a series of gestures, well-spaced in time (Liebal et al. 2004a). From the ape’s perspective, each gesture is a separate attempt to achieve its single goal: a series of this kind is best regarded as a bout. Often, however, apes make several gestures in quick succession at the same target audience, too rapidly for response monitoring to have taken place (Genty and Byrne 2010; Hobaiter and Byrne 2011b). Evidence that this kind of gesturing is genuinely different in kind from mere persistence comes from a study which divided series of gestures according to whether items were separated by >1 s, or by ≤1 s (Hobaiter and Byrne 2011b). In well-spaced bouts of gestures, the gesture type was usually the same, repeated. In rapid-fire “sequences” of gestures, however, much greater variation in gesture type was found: sequences typically consist of several synonyms. What is the purpose of such rapid rotation among gesture types of the same meaning?

In chimpanzees, gesture sequences are given more by young individuals: sequence use declines steadily with age (Hobaiter and Byrne 2011b). One obvious possibility to explain this pattern would be if sequences were given for emphasis: young individuals might need to emphasize their intentions more than older ones. However, no evidence has been found that sequences *are* more effective in evoking a satisfactory response, other things being equal; indeed, single gestures are more effective. Instead, it seems that certain specific gestures are more effective than others, regardless of the age of the signaller. Moreover, when gestures are categorized as “effective” or “less effective” for a specific community of chimpanzees, then the likelihood of choosing an effective gesture increases with age of the signaller (Hobaiter and Byrne 2011b). These facts led to a developmental hypothesis to explain the existence of sequential gesturing. Young individuals, exploring their very large natural repertoire, typically have available several gestures for each purpose: perhaps they do not initially know which one will be most effective, in their community (*ibid*). The option of stringing several of these synonyms together, producing a sequence of varied composition, does at least make it likely that one of the gestures will prove to be an effective one. As they come to learn which gestures are the best to use, the need for sequence use declines. A prediction from this hypothesis is that, in an entirely novel situational context, the same “scattergun” approach would be expected even in an adolescent or adult chimpanzee. One of the chimpanzee’s mating strategies, termed “consortship”, presents just such a test (Tutin and McGrew 1973). In this, a sexually swollen adult female leaves the core area of her community with a single adult male, remaining apart for several days over the peak of her fertile period. The male has no opportunity as a juvenile to experience this situation, and as predicted his gestural communication reverts to sequences, in his efforts to persuade the female to remain with him in an affiliative relationship (Hobaiter and Byrne 2012).

The recorded active repertoire of adults is much lower than that of juveniles, which in turn is larger than that of infants (Call and Tomasello 2007a; Genty et al. 2009; Tanner and Byrne 1999; Tomasello et al. 1989); the hypothesis that young apes experiment with their innately specified repertoire accounts for these differences (Hobaiter and Byrne 2011b). The developing ape first explores its own (potential) repertoire, actively using more and more gestures—often in sequences since it is unsure which single gesture would work best. As it gradually acquires that extra knowledge, sequence use declines and many gestures are no longer used at all, so adult repertoires give the misleading impression of impoverishment. Acquisition is a matter of pruning an innate repertoire, rather than accretion of new gestures.

But have adult apes forgotten those gestures they used to use? The data from what is usually called “gestural imitation” suggest not. In this paradigm, subjects are first taught the command, “do this,” using food rewards with a training set of actions; then novel actions are introduced, and the subject’s behaviour videotaped (Call 2001; Custance et al. 1994; Hayes and Hayes 1952). Naïve coders, shown the recording, are readily able to identify which novel action ape subjects had seen; however, although the copies match the demonstrations, they are often rather a poor match: for instance, a two-handed covering of the ears might be copied with only one hand. These data are usually interpreted as evidence that great apes can imitate arbitrary, novel actions, but there is another possibility. With the extensive repertoires of gestures with which apes are naturally equipped by their biology, the demonstrations might only be priming gestures already in the potential repertoire, but no longer used in adulthood (Byrne and Tanner 2006). This hypothesis can explain why the “copies” were often not very accurate: because they were not copies of novel actions, but rather gestures of the individual subject which had not been used in recent years, brought out by the facilitation of seeing a physically similar action done by the experimenter. To test this hypothesis, a near-complete repertoire for the experimental subject, based on years of painstaking observation, would be required: and exactly that did exist for the gorilla Zura, part of an 11-year study of gesture (Tanner 1998). Zura began spontaneously “imitating” human actions made by the researcher, Joanne Tanner. Tanner chose to demonstrate specific actions she judged would be novel to Zura; but when Zura’s “imitations” were compared against the long-term database of her gestures, every one of them was found to have been used before (Byrne and Tanner 2006). The gestures Zura had long ago performed spontaneously were what she produced in response to the demonstrations, not imitated copies of what she was shown: so, they were often slightly different, as with other reports of gestural imitation. The priming of rarely used items in a very extensive repertoire may therefore explain the behaviour of all the great apes that have shown “gestural imitation”, implying that the gestures explored and discarded by apes during the process of growing up are not lost, but remain in their passive gestural repertoire. Presumably, the apes remain aware of the meaning of the gestures and would recognize them if the gestures were used by others, even though they no longer use them themselves.

## Conclusions

There is undoubtedly much more to discover about ape gesture, but what we currently know paints a puzzling picture. Apes give gestures deliberately and voluntarily, in order to influence specific target audiences, whose direction of attention they clearly appreciate and take into account when choosing which type of gesture to use. Compared to human words, the meanings that a signaller intends to convey by using gestures are relatively few and simple. As in the case of words, however, ape gestures often have several distinct meanings, which are largely disambiguated by situational context. Thus, the real size of an ape’s repertoire must be greatly underestimated by counting gesture forms, as done at present. Because of the large size of the repertoire and the relatively small number of intentional meanings it is used to achieve, ape gestures are redundant. Most surprising of all, perhaps, is gesture ontogeny. No doubt, occasionally, apes do add idiosyncratic action patterns to their gestural repertoire by the mutual conditioning within a regular dyad that has been termed ontogenetic ritualization; but this is apparently much rarer than was once thought, and in extensive field studies of gesture in chimpanzees and bonobos there was no evidence for it at all. No doubt, occasionally, a local tradition of using a gesture may develop, unique to a single population, but this again appears relatively infrequent. The great majority of gestures in the ape repertoire—and that is a large number, compared to that of most other animals—are innate, in the sense that the potential to develop a particular gestural form and use it for a particular, restricted range of purposes is part of the species’ biological inheritance. Moreover, the phylogenetic origin of many gestures is relatively old, since the gesture forms are extensively shared between different genera in the great ape family (and their meanings are the same across species, at least within *Pan*). Young individuals, apparently unsure of which gestures will be most effective for their purpose, use several equivalent gestures and thereby generate rapid-fire sequences of gestures. As they gain experience, they increasingly pick the most effective single gestures: usage learning occurs by pruning, as found in human phonemic development. Adults, as a consequence, use fewer gesture types than young animals and rely on sequences less. Acquisition of an adult repertoire is a process of first exploring the innate species potential to use a large number of gestures, then gradual restriction to a final (active) repertoire that is much smaller. Adults have not apparently forgotten their full repertoire of gestures, because their latent repertoire can be revealed experimentally. In “gestural imitation”, gestures from this extensive latent repertoire are facilitated. Because the copies are in fact part of the individual’s own repertoire, the match to the demonstrations is often not perfect.

We are left with a puzzle. We know that great apes can readily learn novel manual gestures, as is shown most obviously in the “ape language” studies of home-reared apes (Fouts et al. 1989; Gardner and Gardner 1969; Gardner et al. 1989; Miles 1990; Patterson and Linden 1981). So why don’t they use this ability to augment their natural gestural repertoires in ways appropriate to their individual ecology and social circumstances? It seems possible that great apes’ innate repertoire is so extensive that they never reach the point at which they need to communicate something more: and indeed gestures are redundant, so that if further differentiation of meaning were needed, the gesture forms are already available to be co-opted. This explanation suggests a surprising lack of imagination. We might draw an analogy with a situation familiar to many infant teachers: two children learning to read and write. One, who is dyslexic but bright and highly motivated, has difficulty mastering the mechanics of the process, but really benefits from the reading and writing they can achieve; the other soon learns the techniques, but doesn’t really see the point of reading, let alone writing, because of lack of imagination. Perhaps the restricted communication of great apes stems from a general limit on their imagination, rather than a specific block on using gesture to communicate?
